# An advanced YOLO-based image processing framework for automated sperm cell detection

**DOI:** 10.1038/s41598-026-50401-9

**Published:** 2026-05-19

**Authors:** L. Prabaharan, A. Sivapathi, L. Gowri

**Affiliations:** School of Computing, SASTRA Deemed to be University, Thanjavur, Tamil Nadu India

**Keywords:** Biological techniques, Computational biology and bioinformatics, Engineering, Mathematics and computing

## Abstract

Detection of sperm cell is an extremely important procedure in medical diagnostics and fertility research. Given the need to identify sperm in an efficient manner, this paper offers a powerful image processing pipeline. This method starts with image grayscale conversion, which is used to simplify the image and reduce the complexity of color, which is followed by Gaussian blur and Wiener filter to remove noise and improve image quality. An optimal-threshold is obtained by thresholding to obtain the sperm cells out of the background with the binarization method of Otsu which ends by concluding. Alternatively, adaptive Havrda-Charvat entropy thresholding is also discussed to allow better accuracy under difficult circumstances, correcting to differences in the intensity of the image. The sperm separation is then carried out in the segmentation phase, in which the sperm cells are segregated out of non-relevant objects. The last one is an automatic system that can identify sperm cells with high accuracy, which allows quicker and more accurate analysis by deep convolutional neural network of YOLOv5s to be used in clinical and research applications. The offered technique is tested with the help of a collection of microscopic images, which proves its efficiency under various light conditions and morphological changes of sperm.

## Introduction

Male infertility is brought about through sperm cell abnormalities in approximately 30% of infertile couples. The traditional techniques of assessing the sperm morphology are tedious and rely on the judgement of the technician. The versatility of digital imaging and image processing procedure has given rise to the curiosity of development of automated and objective procedure of sperm morphology analysis. This section provides an overview of the recent improvements in image processing algorithms to detect abnormality of the sperm cells. The most common problem exhibited by married couples is infertility of which one in ten couples is infertile. It creates psychological as well as social problems^1^. A detection of spermatozoon cells is vital in the reproductive medicine, diagnostics, and fertility research. The reliable counting of sperm is used to determine or examine the fertility of males and morphology of the sperms which may be used to determine treatment or to conduct further diagnostic tests. With automated sperm cell detection, the analysis is more because it is speedier and reliable compared to manual identification that is human error-prone and labour-intensive. Seminal examination is one of these and this is critical in determining the number of sperms, their morphology as well as their motility. Nevertheless, the conventional approaches based on manual microscopy are man power demanding and will be subject to variations in observation and thus their reliability is restricted^2^.

**Fig. 1:**
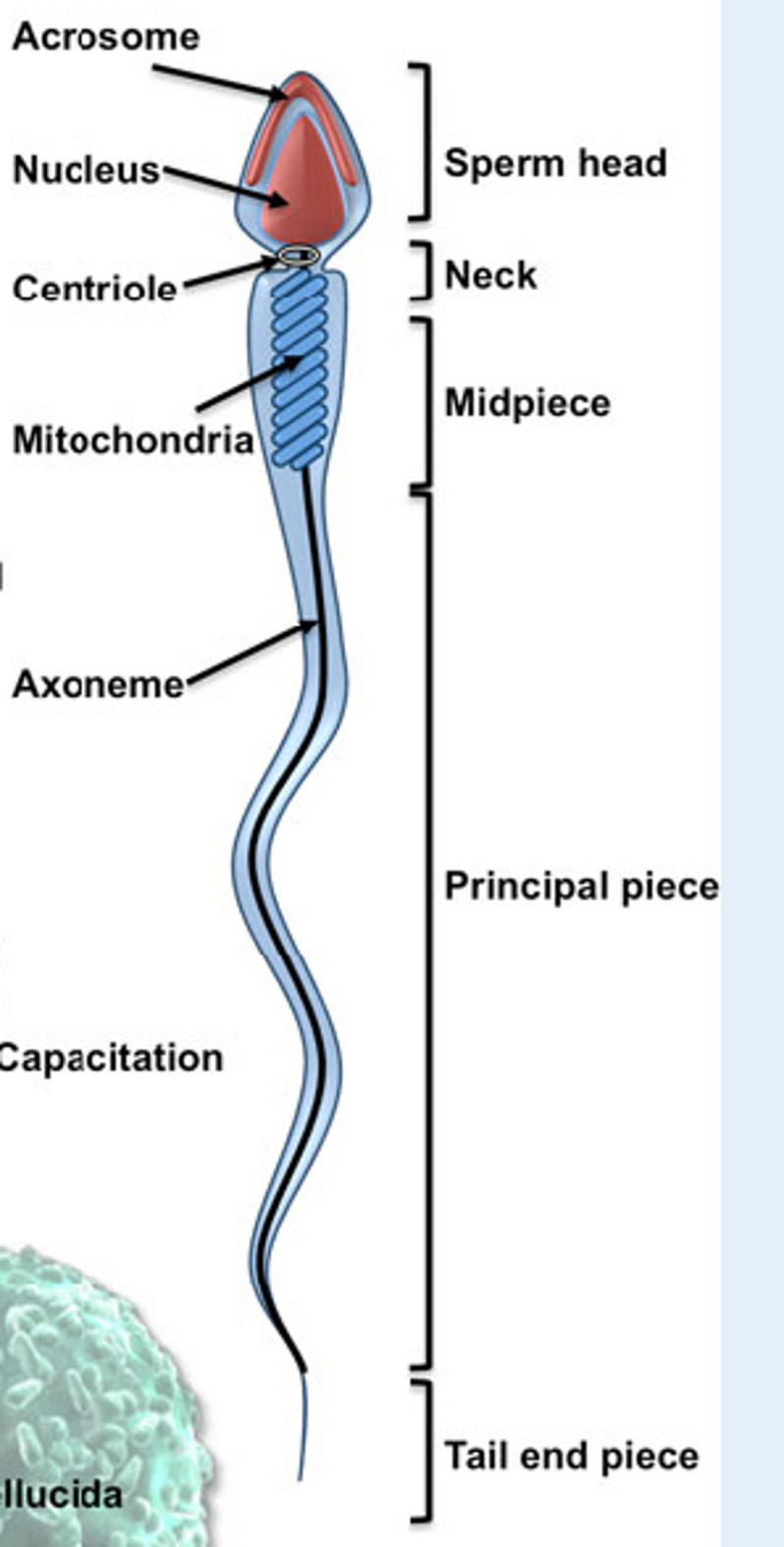
Schematic diagram of sperm cell with acrosome, nucleus, centriole, mitochondria, axoneme and tail (head, neck, midpiece, and principal piece). OpenStax based on human anatomy.

More recently, automated methods have been developed such as Computer-Aided Sperm Analysis (CASA) in order to overcome such limitations. CASA systems make sperm analysis more accurate because they utilize sophisticated algorithms, but there are weaknesses, such as a high price and sensitivity to the quality of images^3^. The noise is removed and the image visual quality is enhanced with the aid of pre-processing techniques^4^. The sperm cells in the selected focused area were divided into parts through a mid-level process and their head, middle-piece, and tail were determined through the application of a high-level process (see Fig. 1 to get a schematic view of the same). Noise and additives may be enhanced by the use of electronic imaging tools and unadvisable processes^5^. Hence, noise-free cell image has to be developed. Image denoising is an image processing method to remove unwanted details on the images when capturing them and when scanning them. Noise subtraction is a more challenging pre-process that needs to be carried out to preserve even little details in cell-image. The choice of a noise-reduction filter assists in the removal of obstructive images to the extraction of ROI because it saves the fine details of the image. In spermatozoon cell-image processing, it is important to have better de-noising methods in order to retain minute details of an image. The many staining procedures used in the generation of images normally produce cloudy images of the cells. Many of the recently created noise-removal filters are very well explored in terms of noiseless image^6^ due to their pioneer creation in development of microscopic medical imaging.

Image-segmentation refers to the procedure of obtaining relevant data^7–9^ of an input-spermatozoon cell image to more analysis. The tail-body-head sections of spermcell may be focused using input-image in an attempt to study sperms cell abnormalities. Segmentation method can be further classified into three namely region segmentation^10^, boundary segmentation^11^, and thresholding^12^. Boundary based segmentation is done on the basis of contours between regions. The Sobel technique^13^ is widely used edge detection, basis of gradient value variation^14^. The regional segmentation technique is based on area similarities. A picture is divided by the region expanding method which blends the adjacent pixel is based on the matching criteria^15^. Sperm analysis has also been widened out by image segmentation techniques which can be used to extract sperm cells out of complicated backgrounds. Conventional methods, including thresholding and edge detection, are typically accompanied by machine-learning model includes convolutional neural networks (CNNs). Such methods enhance better segmentation, particularly those concerning the situations where neighboring sperm cell are very close to each other^16^. Recently, deep learning architectures, such as modified U-Nets, have demonstrated high prospects in medical image -segmentation. The models of this type are able to outline the cells in the microscopic image successfully, despite such difficulties as poor quality of the image and the overlapping of objects. Through augmentation with image transformation these frameworks are designed to increase the strength and flexibility in clinical settings^17^. You OnlyLookOnce (YOLO) deep-learning based object identification algorithms that have gained extreme popularity due to its precision and real time speed in application. During one forward pass of a neural network, single regression has been used to solve an objectdetection problem in YOLO, which literally results in the direct prediction of bounding box positions and probabilities of being a specific object (class) based on a direct image^18^. Recent studies have discovered that YOLO can be applied in biomedical applications in the tracking of microorganisms, cancer cell detection, and counting. It has also been pointed out as effective in sperm cell identification^19,20^. YOLO can pick even tiny and irregularly formed objects in the compromised imaging conditions due to its structure which is composed of numerous convolutional layers and anchor box techniques. By training YOLO models on annotated microscopic sperm datasets, researchers are capable of doing automated, repeatable, and high-throughput sperm detection which facilitates more reliable clinical assessment.

To successfully detect sperm cells, we introduce in the present study a YOLO-based Sperm pipeline which process microscopic images to detect sperm cells. Annotated sperm cell datasets are publicly available and are used to train and evaluate the model. We also investigate the advantages of YOLO in matters of accuracy, speed, and also strength with different imaging environments and also compared its execution with conventional image processing methods.

This paper is dedicated to the creation and implementation of image enhancement and segmentation algorithms that are specific to sperm, which should help to provide proper detection and description of sperm cells with the help of YOLO. In solving the existing constraints, it aims to advance enormously to the diagnostic modalities of reproductive medicine. Figure 1 has represented the schematic-diagram of sperm-cell. The subject of interest in the sperm cell is the morphological analysis through which it is possible to identify normal sperm cell in order to use it in facilitating the method of fertilization.

### Novelty and contributions of the proposed work

Though a number of studies have been conducted on sperm cell detection with the classical image processing or with the deep learning models, most current methods involve only with the conventional segmentation algorithms, or simply utilize deep learning models on raw microscopic images. These techniques are usually weak in the presence of noisy images, uneven illumination and complex microscopic backgrounds. To solve these shortcomings, this research paper suggests a combined multi-step method that integrates adaptive image enhancement, entropy-based segmentation, and object detection based on deep learning to achieve a dependable method of sperm cell identification.

The most important works of this piece are summarized as follows:

1. Deep learning framework- hybrid image processing.

It also suggests a three-step model that combines image enhancement, conventional segmentation, and object detector-based on the YOLOv5 model to enhance the accuracy of the sperm cell detection in microscopic images.

2. Adaptive dual-threshold-segmentation strategy.

This framework applies an adaptive segmentation algorithm enabling a switch to an Otsu binarization or adaptive HavrdaCharvat entropy thresholding as the image variables vary to offer a more favorable segmentation under various conditions of environment illumination and contrast.

3. Guided deep learning detection: this involves a pre-processing stage.

In contrast to most other available YOLO-based methods that perform their tasks directly with raw microscopic images, the method proposed is accompanied by organized pre-processing on Gaussian and Wiener filtering to alleviate noise and fine morphological features prior to detection.

4. Strengths of detection in imaging variability.

The framework demonstrated a high level of performance by providing consistent detection in varied environments with respect to illumination and morphological variation of sperms and high levels of precision and recall within experimental assessments.

5. Automated semen analysis: practical applicability.

The suggested pipeline is computer-efficient and can be integrated into Computer-Assisted Semen Analysis (CASA) systems to allow quicker and objective sperm morphology evaluation in the clinical and laboratory environments.

Based on these contributions, the suggested solution will help to increase the accuracy of automated sperm cell detectors and improve the creation of smart diagnostic methods in the field of reproductive medicine.

## Literature review

The authors of^21^ propose the automatic sperm image detection method. In this case, aberrant semen smears of human semen are detected through the use of semen smears. Human semensmears of sperm head, acrosome and nucleus under a microscope. Thresholding is first done in histogram equalisation and hue channel is employed in marking the mask. Mapping of the sperm’s head is done using segmentation method which can utilizes the edgebased active-contour technique. Morphological operations such as segmentation of the midsection of the sperm head and height and width of sperm are performed using dice coefficient to determine aberrant sperm images. Acrosome-nucleus is destroyed to bridging head-contours. Carrillo method assumes abnormalities then by means of edge detecting and counting techniques. Precision and F-score values of this image are compared with relevant methods available.

The experts of^22^ focus segment-process, accurate definition of multiple portions of the sperm, and detailed research of the characteristics of the head. The input of the algorithm is an image. The authors were able to determine the head of sperm as high as 98% in a twenty spermImage dataset.

In^23^, the authors report a computer-based technique of the sperm-movement and semen-concentration. It proposes neat-algorithm to enhance a segmentation technique, geometric features to identify and analyze sperm cells in a microscopic image. Speed, precision, complexity of method is evaluated.

The scientists of^24^ have outlined a method of morphological detection of anomalies in human sperm photos by automated methods. Morphological study is the primary step of diagnosing male infertility-pattern in humans. SMA algorithm is based on structural -dimensions and size of head-tail can determine good cells. Intra-cytoplasmic sperm injection is the method of real time detection of the best cells by the embryologists. First of all, the input-image is processed with medianfilter to locate head, neck-tail and examines vacuole and head-size to categorise sperm-head followed by a Gaussianfilter to refine color channel. The way that noise has been reduced is by using the wavelet-transform and reduction of the edge has done by using the sobel expression. Head recognition-phase, is a very crucial study to locate abnormalities. Comparing the approach to other techniques, accuracy and computation-time are compared.

To eliminate such image segmentation issues, an effective deep learning framework^25,26^ is required. The colossal capabilities of these methods in the analysis of biomedical images lie in the speed and efficiency with which they perform their job. It can be applied to various tasks, such as segmenting ad identifying objects^27,28^. Not all developments have been utilized fully. The specialised field is not comparable to the old methods and most recent deeplearning methods^29^.

The authors^30^ propose the multi-feature method of diagnosing the sperm abnormalities using SVM. In this case a morphological method is employed in order to quantify a one dimensional property. The morphological procedure is done depending on the general well being of the sperm cells. In this case, the SVM way of doing the classification becomes more accurate. In order to carry out segmentation, the sperm head is pointed out. In this case, bilateral symmetric strategy was proposed to be applied in continuous studies of sperm cells.

The author of this review^31^, explores various deep learning processes of spermcell segmentation and fertility-prediction, highlighting how traditional image processing methods have been replaced by advanced deep learning systems, including CNNs.

The scholars examined sperm cell detection^32^. The article presents a computer-assisted algorithm of sperm detection with the help of YOLOv5. Creation of labelled-datasets has been highlighted, by which the model can enhance its ability to detect sperm cells in small size images.

Authors of^33^ optimized UNet on the Sperm-head segmentation task by using a new dataset, specifically built to be used in deep learning, and explored the model, which showed better results on the distinction of sperm cells and non-sperm objects.

The authors of^34^ propose the use of the supervised machine-learning methodology to predict spermcell abnormalities. Magnetophoresis of the sperm are done using the thin body theory. This is a process that determines the statistical variance of spermatozoa. This technique currently being used evaluates four monitored-machine learning models that predict aberrant sperms. In this case, machine learning training process is combined with the calculation of the slender body theory. The approximation of an estimated calculated value is taken to train and finding the anomaly.

This paper is organized as follows: starting with literature review, followed by a three steps framework and concluded with results and discussion.

## Methodology

**Fig. 2:**
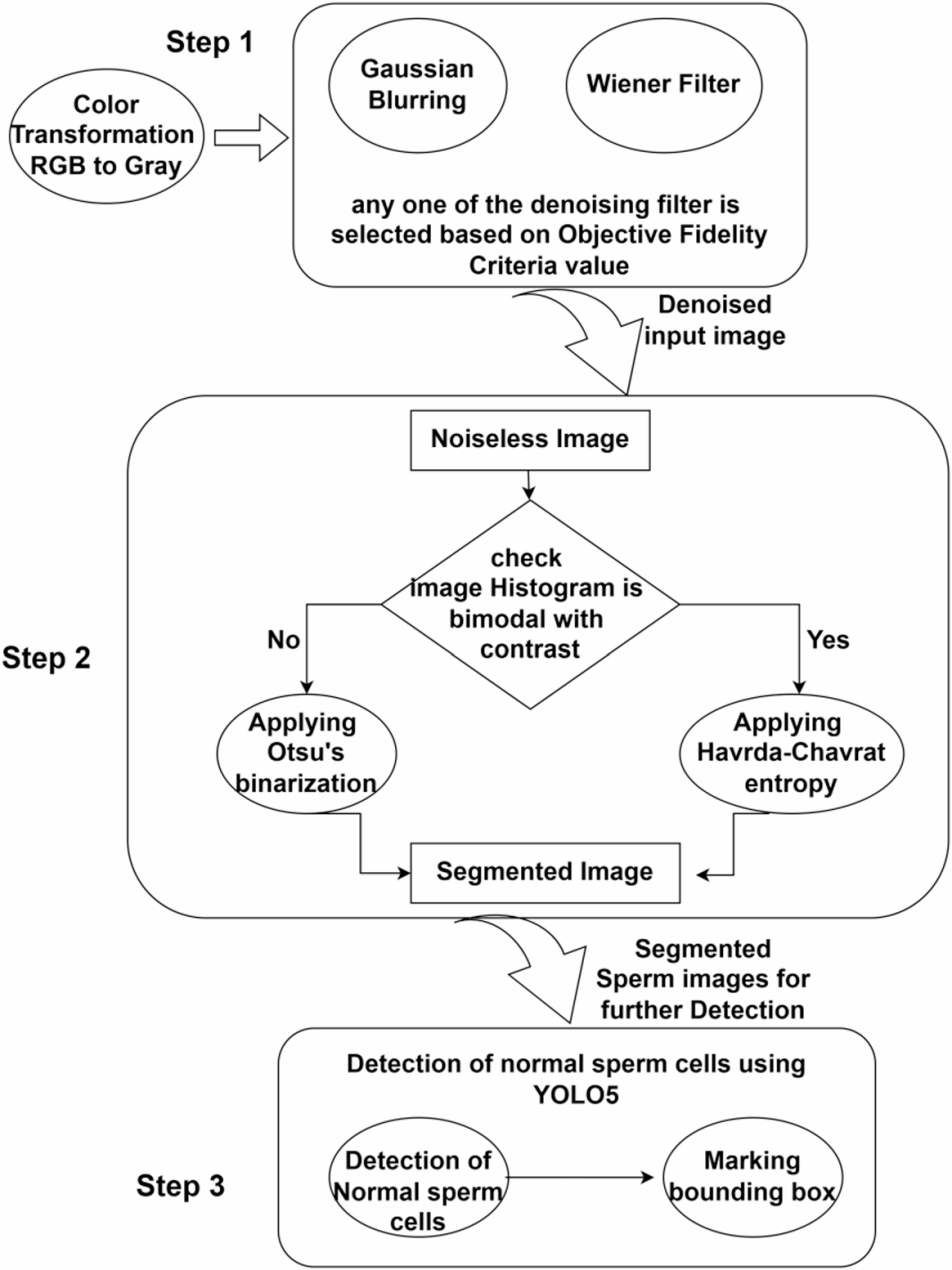
An overview of an advanced YOLO-based image processing framework.

Preprocessing directed detection method is adopted to improve the detection strength in microscopic sperm images. The given process comprises three key stages: (i) transformation and de-noising of the images (ii) segmentation of images with the help of classical thresholding procedures, and (iii) detection of objects with the help of a deep learning algorithm called YOLOv5, but specifically trained to detect morphologically normal sperm cells. Figure 2 shows the overview of proposed framework. This is done in a detailed methodology as follows:

The image dataset employed in this work was collected from the publicly available web source^35^, which is widely recognized for its contribution to sperm cell image segmentation studies. The dataset is considered gold-standard with respect to sample preparation and image acquisition quality. It consists of 19 stained microscopic images of spermatozoa samples containing 264 sperm cells in total. Since the original dataset size was limited, data augmentation was performed to expand it to approximately 10,000 images for effective YOLOv5 training. The final dataset was split into training, validation, and testing sets in an 80:10:10 proportion, corresponding to approximately 8,000, 1,000, and 1,000 images, respectively. Prior to training, all images were resized, normalized, and converted into YOLOv5-compatible annotation format. Augmentation operations, including rotation, horizontal/vertical flipping, scaling, translation, brightness and contrast adjustment, and noise addition, were applied only to the training subset, whereas the validation and test subsets underwent only resizing and normalization.

### Step 1: Image improvement, colour transformation and de-noising

#### Grayscale conversion

**Fig. 3:**
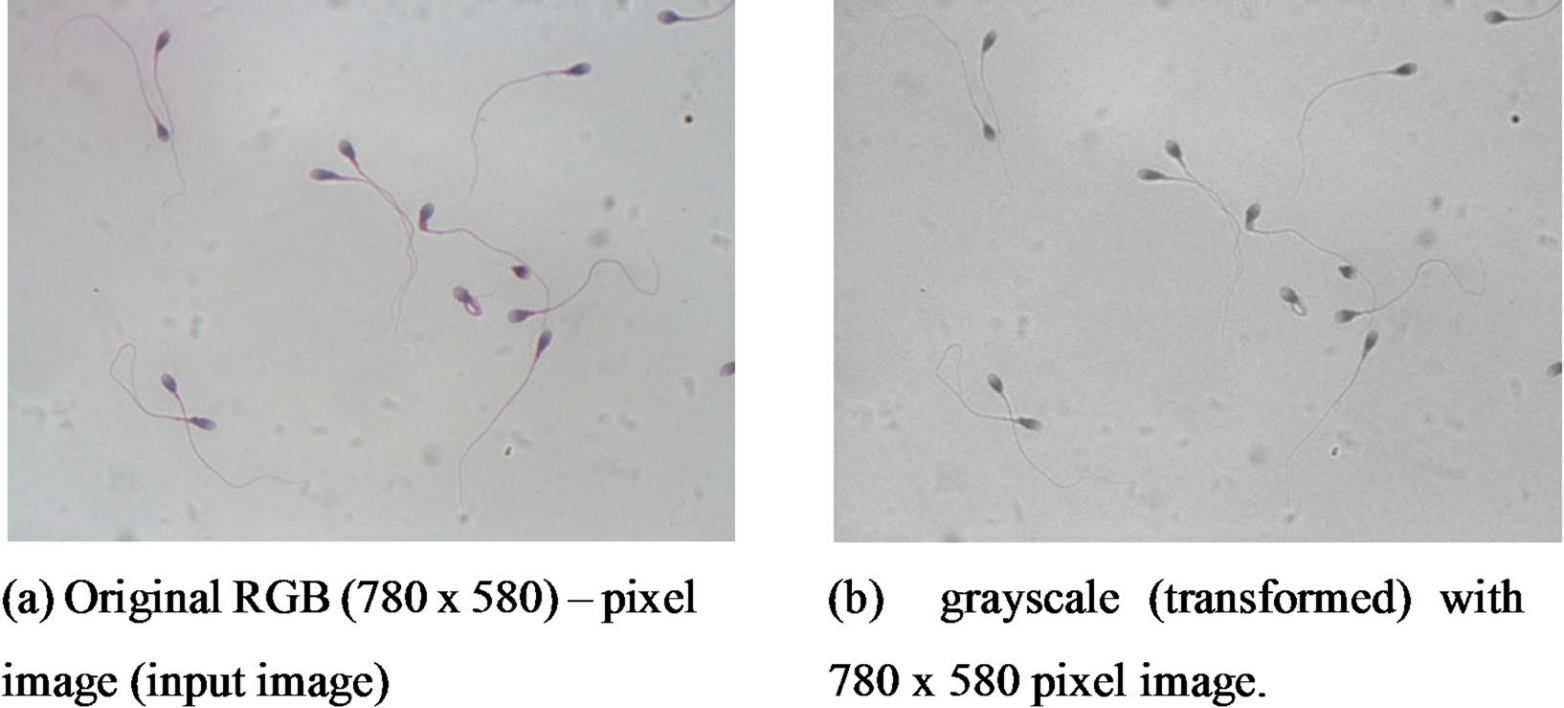
Conversion of an input picture which is in RGB color space to Gray Scale.

The complexity of the color of the microscopic image decreases by converting RGB image to the grayscale image. The simplification is essential since it eliminates the redundant color data and narrows down to differences in intensity. The transformation of color can be written as shown in Eq. (1) below. Image is then transformed to image that is further processed with noise removal which is elaborated in step 2. Figure 3 provides the sample of input images.

1 $$\:\frac{\mathrm{max}\left(R,\:G,\:B\right)+\:\mathrm{m}\mathrm{i}\mathrm{n}(R,\:G,B)}{2}$$

#### Wiener de-noising or Gaussian blurring

Gaussian blurring is used to minimize high-frequency noise and enhance the capability of edge detection. This action helps to prevent transition difficulties by eliminating small pixel-level noise that might disrupt thresholding and contour detection. Image is blurred with linear_filter and high frequency values are passed (lowpass filter). Rather than being effective with removing impulse noise, the filter removes Gaussian-blur in color transformed image. The Gaussian filter is provided in the Eq. (2).

2 $$g(x,y)=\frac{1}{{\sqrt 2 \pi {\sigma ^2}}}{e^{ - \frac{{{x^2}+{y^2}}}{{2{\sigma ^2}}}}}$$

Based on the objective measures of fidelity such as MSE (Mean Square Error), (PSN) Peak Signal to Noise Ratio, Kurtosis and Entropy applied on blurred image after Gaussian blurring, it is determined whether the noise is removed or suppressed. Table 1 displays the objective fidelity criteria value. In case the noise is not eliminated according to the objective fidelity criteria value, the image is subject to Wiener filter (3). Wiener filter is effective in undoing the blurring in image and remove additive error. This filter takes into account the linear approximation of an image with the help of a stochastic method. Wiener filter gives the image a smooth look and reduces the mean square error.

3 $$W(u,v)=\frac{{H*(u,v){s_{xx}}(u,v)}}{{{{\left| {H(u,v)} \right|}^2}{S_{xx}}(u,v)+{s_{\eta \eta }}(u,v)}}$$

Noisy Image has been applied with respective filters in the order as shown below Comparison of noisy and Filtered Image.

**Table 1:** The (OFC) objectiveFidelity criteria for the Filtered Images.

Noisy Image applied with following filters	Comparison of noisy and Filtered Image		
	MeanSquare Error	Peak Signal to Noise Ratio	Entropy
Gaussian filter	90.79	28.55	3.55
Wiener-filter	55.77	30.66	3.50

### Step 2: Base Level 2 Imagery segmentation classical thresholding

The process of isolation of sperm cells against the background depends on thresholding. There are two methods of thresholding:

#### Otsu binarization

It is clustering threshold method, in which the image is divided into two divisions (classes) to minimize the distinctions within each division, is among the most commonly mentioned clustering thresholding approaches^36^. When threshold-value is chosen, the breadth distribution changes in the 2 groups. Selection of threshold narrows down widths of two distributions. The suited threshold for segmentation process is computed by calculating the intraclass and interclass variances as in the equation between (4) and (8). The interclass variance is computed by subtract the intraclass variance of the total variance as shown in Eq. (4).

4 $$\:{{\sigma\:}^{2}}_{\mathrm{i}\mathrm{n}\mathrm{t}\mathrm{r}\mathrm{a}}\left(\mathrm{T}\right)={\mathrm{n}}_{\mathrm{B}\mathrm{G}}\left(\mathrm{T}\right){{\sigma\:}^{2}}_{\mathrm{B}\mathrm{G}}\left(\mathrm{T}\right)+{\mathrm{n}}_{\mathrm{F}\mathrm{G}}\left(\mathrm{T}\right){{\sigma\:}^{2}}_{\mathrm{F}\mathrm{G}}\left(\mathrm{T}\right)$$

Where

5 $$\:{n}_{BG}\left(T\right)={\sum\:}_{i=0}^{\mathrm{T}-1}\mathrm{p}\left(\mathrm{i}\right)$$

6 $$\:{n}_{FG}\left(T\right)={\sum\:}_{i=T}^{\mathrm{N}-1}\mathrm{p}\left(\mathrm{i}\right)$$

7 $$\:{{\sigma\:}^{2}}_{\mathrm{B}\mathrm{G}}\left(\mathrm{T}\right)=\mathrm{T}\mathrm{h}\mathrm{e}\:\mathrm{v}\mathrm{a}\mathrm{r}\mathrm{i}\mathrm{a}\mathrm{n}\mathrm{c}\mathrm{e}\:\mathrm{o}\mathrm{f}\:\mathrm{b}\mathrm{a}\mathrm{c}\mathrm{k}\mathrm{g}\mathrm{r}\mathrm{o}\mathrm{u}\mathrm{n}\mathrm{d}\:\mathrm{p}\mathrm{i}\mathrm{x}\mathrm{e}\mathrm{l}\mathrm{s}.$$

8 $$\:{{\sigma\:}^{2}}_{\mathrm{F}\mathrm{G}}\left(\mathrm{T}\right)=\mathrm{T}\mathrm{h}\mathrm{e}\:\mathrm{v}\mathrm{a}\mathrm{r}\mathrm{i}\mathrm{a}\mathrm{n}\mathrm{c}\mathrm{e}\:\mathrm{o}\mathrm{f}\:\mathrm{f}\mathrm{o}\mathrm{r}\mathrm{e}\mathrm{g}\mathrm{r}\mathrm{o}\mathrm{u}\mathrm{n}\mathrm{d}\:\mathrm{p}\mathrm{i}\mathrm{x}\mathrm{e}\mathrm{l}\mathrm{s}.$$

9 $$\:{{\sigma\:}^{2}}_{\mathrm{i}\mathrm{n}\mathrm{t}\mathrm{e}\mathrm{r}}\left(\mathrm{T}\right)={{{\sigma\:}^{2}-\sigma\:}^{2}}_{\mathrm{i}\mathrm{n}\mathrm{t}\mathrm{r}\mathrm{a}}\left(\mathrm{T}\right)$$

**Fig. 4:**
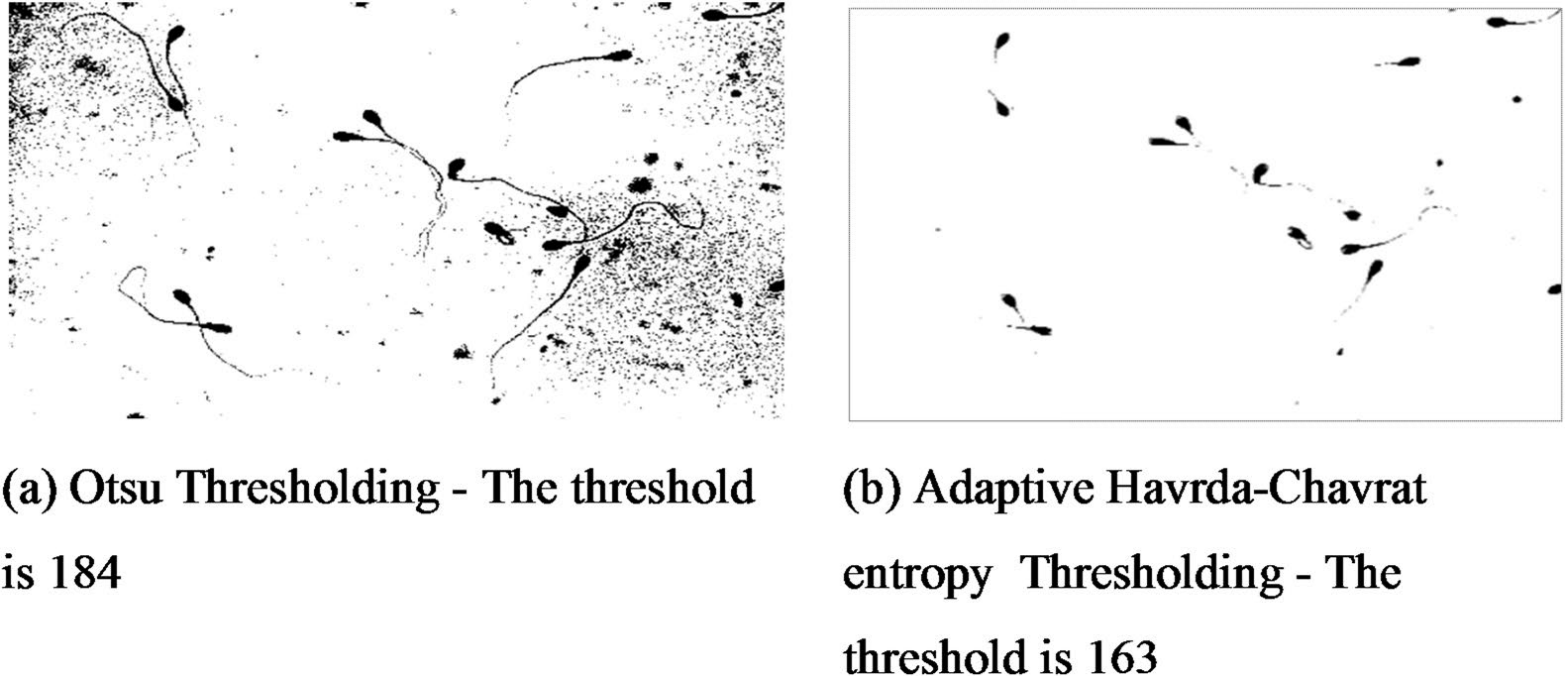
Segmented image.

#### Entropy thresholds: adaptive Havrda-Charvat

Here, threshold is adjusted based on the local changes in signal intensity^37^, a feature that is especially effective when the input image has different lighting or noise conditions Input_ image, f(a, b) prepared with spatial -resolution (M x N) and grayscale (0, n) is separated by entropy-based segmentation methods. The intensity values whose pixels have t range^1^ are split into 2 divisions X, Y. The rest of the pixels in division Y are the ones that take values bigger than t. Threshold is the separation point in the histogram. There are 2 probability distributions for image histogram, namely X and Y, one of them corresponding to the object and the other to the backdrop. The alpha (a) parameter is a non-zero number between 0 and 1, such that an ideal threshold in this entropy-based thresholding method is given by alpha(a) value. Alpha is one of the important factors that influence the thresholding result. A greater alpha normally gives a lower threshold that may include background to the item. There is the risk that the segmentation does not pick up the object in case the threshold is increased by a smaller alpha. The alpha value is manipulated manually through trial-and-error to find the best threshold. With proper choice of alpha, the method yields the best segmentation as shown in the Eqs. (10) and (11).

10 $${}_{{Hav}}\psi _{x}^{\alpha }t={\left( {{2^{1 - \alpha }} - 1} \right)^{ - 1}}\sum\limits_{{i=0}}^{t} {\left[ {{{\left( {\frac{{{p_i}}}{{{P_t}}}} \right)}^\alpha } - 1} \right]}$$

and

11 $${}_{{Hav}}\psi _{y}^{\alpha }t={\left( {{2^{1 - \alpha }} - 1} \right)^{ - 1}}\sum\limits_{{i=t+1}}^{n} {\left[ {{{\left( {\frac{{{p_i}}}{{(1 - {P_t})}}} \right)}^\alpha } - 1} \right]}$$

The threshold value is not an absolute value and is associated with the level of gray of the pixel. Entropic thresholding is computed using the probability distribution of image intensity which is used to describe the texture of the image. The optimum entropic threshold value is determined by considering the maximum probability distribution of the foreground or the background pixels.

The input image is used to determine the choice of alpha value. It is indicated that the likelihoods of the input image foreground and background pixels are procured to compute the alpha value. The above image f(x, y) with spatial resolution of M x N and the intensity of each pixel I with the grayscale [0, n] that is partitioned into 2 distinct distribution groups, d1 and d2, according to the statistical mean such that division d1 is the pixels with the intensity values in the range of [0, mean]. Division d2 has pixels with intensity value not found in division d1 in division d2. The probability of all pixels in the division d1 and d2 should not be one. This distribution is able to determine the significant pixels in obtaining an alpha value. The alpha value extraction process of the input image is as follows:

(i). Find the probability density of the input image f(x, y) by the number of pixels totaling with the sum of the frequency of each range of possible pixels.

(ii). Divide the input picture f(x, y) with spatial resolution M x N and bit-level resolution k with grayscale [0,L-1] by L = 2k to obtain the mean (‘M’) of the input picture as follows

12 $$\:\mu\:={\sum\:}_{x=0}^{L-1}\mathrm{x}.p\left(\mathrm{x}\right)\:\{\backslash\:\mathrm{d}\mathrm{i}\mathrm{s}\mathrm{p}\mathrm{l}\mathrm{a}\mathrm{y}\mathrm{s}\mathrm{t}\mathrm{y}\mathrm{l}\mathrm{e}\:\backslash\:\mathrm{t}\mathrm{e}\mathrm{x}\mathrm{t}\mathrm{s}\mathrm{t}\mathrm{y}\mathrm{l}\mathrm{e}\:\backslash\:\mathrm{s}\mathrm{u}\mathrm{m}\:\mathrm{x}\mathrm{P}(\mathrm{x}\left)\right\}$$

where $\:\mathrm{P}\left(\mathrm{x}\right)$ denotes probability of pixels and (‘µ’) is mean.

(iii). With the help of mean (‘µ’), segment the input image $\:f(x,y)$ into 2 separate parts using the foreground pixels, which accommodate the intensity values, which are less than or equal to mean (‘µ’) and the background pixels, which comprise the intensity values, which are greater than mean intensity.

13 $$d1=\sum\limits_{{i=0}}^{\mu } {p(i)}$$

and

14 $$d2=\sum\limits_{{i=\mu +1}}^{{L - 1}} {p(i)}$$

(iv). Compare the overall distribution of pixels of $\:d1\:and\:d2$

15 $$\:\genfrac{}{}{0pt}{}{\begin{array}{c}\left\{\begin{array}{c}d1\\\:d2\end{array}\right.\:if\:\:\left(d1\ge\:d2\right)then\:\:\:\:\:\:\:\:\:\:\alpha\:=d1\:\\\:\alpha\:=d2\:\:\:\:\:\:\:\:\:\:\:otherwise\:\:\:\:\:\:\:\:\:\:\:\:\:\:\:\end{array}\:}{\:}$$

The automated modified alpha value $\:\left({\prime\:}\alpha\:{\prime\:}\right)$ was obtained by use of Eq. (15) and that was entered into (13) and (14) and obtained.

16 $${}_{{Hav}}\psi _{x}^{\alpha }t={\left( {{2^{1 - \alpha }} - 1} \right)^{ - 1}}\sum\limits_{{i=0}}^{t} {\left[ {{{\left( {\frac{{{p_i}}}{{{P_t}}}} \right)}^\alpha } - 1} \right]}$$

and

17 $${}_{{Hav}}\psi _{y}^{\alpha }t={\left( {{2^{1 - \alpha }} - 1} \right)^{ - 1}}\sum\limits_{{i=t+1}}^{n} {\left[ {{{\left( {\frac{{{p_i}}}{{(1 - {P_t})}}} \right)}^\alpha } - 1} \right]}$$

respectively.

The entropy of this method is maximized and on the basis of the Eq. (15) optimum segmentation is achieved. Using the calculated alpha value, the Havrda-Chavrat entropy equation is used to arrive at the expected threshold value in order to continue with the next level of segmentation. The reason why it is called adaptive is that instead of being assigned a fixed value, the alpha value is calculated automatically based on the input image by examining the probability distribution of the pixels in it.

The above thresholding techniques are very appropriate in segmenting images of microscopic sperm cells. Under the presented algorithm, the thresholding can be Otsu, or adaptive Havrda-Chavrat entropic thresholding. The goal of this algorithm is to get the best threshold that can be used to either minimize intraclass variance or to maximize interclass variance and at the same time maximizes the sum of entropies. According to this criterion, this algorithm suggests either using Otsu threshold or the adaptive Havrda-Chavrat entropy method. The method of Otsu is used when the image histogram is bimodal and the image has good contrast but when the histogram of an image is not significantly bimodal or when the image is noisy, then the method of Havrda-Chavrat is used. The fragmented pictures are represented in the Fig. 4. Figure 4a is an image segmented with the help of the thresholding technique developed by Otsu and Fig. 4b is an image segmented with the help of adaptive Havrda-Chavrat entropy threshold technique. Using the evaluation metrics of segmentation such as entropy, DWR and RAE error, the adaptive Havrda-Chavrat entropic threshold has produced significant values to this provided input image (presented in Table 2). The given method was constructed in such a manner to divide the images with the help of Otsu method or Adaptive Havrda-Chavrat method.

**Table 2:** Segmentation results based on Supervised and Unsupervised metrics.

Segmentation-Detail					Unsupervised metrics			Supervised metrics	
Image	Entropic-thresholding	Threshold	Fore ground Pixels	Background Pixels	D_WR_ (minimum value)	Entropy (maximum value)	NU (close to zero)	ME Error	RAE Error
Figure 3b	Otsu’s threshold	184	2832	449,568	8,372,099	0.073	0.024	0.006	0.395
	The proposed method of segmentation	163	4158	448,242	8,356,866	0.090	0.033	0.004	0.334

#### Reasons as to why preprocessing and segmentation are implemented before YOLOv5 Detection

Even though it is true that modern object detection models like YOLOv5 have the capability of learning discriminative features directly using raw images, microscopic sperm images also have various challenges that adversely impact the learning process. These images usually have background debris, unequal light, staining artifact, and high frequency noise which hide fine morphological features of sperm cells. Also, sperm cells are minuscule entities whose structural content is subtle, which complicates them to distinguish themselves among the background using the raw microscopic images directly as the input of the deep learning-based object detector.

In order to solve these problems, the suggested framework implements a preprocessing and adaptive segmentation phase before the YOLOv5 detection module. The main goal of the step is to improve the appearance of the microscopic images as well as to minimize the irrelevant background structures prior to extracting the features using the deep neural network.

First, noise is reduced by use of Gaussian filtering and Wiener filtering without destroying the significant morphological features of sperm cells. Such filters enhance both the signal-to-noise ratio and enhance the image of the sperm head and tail without distorting the structural features of the sperm.

After noise is disrupted, an adaptive segmentation strategy is implemented to extract the possible sperm areas against background. The segmentation process dynamically chooses either Otsu thresholding or HavradaCharvat entropy-based thresholding depending on the features of the histogram of the input image. This adaptive selection enhances the performance of segmentation in changing illumination conditions and contrast conditions which is usually seen in the microscopic imaging.

This segmentation of the output shows the candidate sperm regions and inhibits irrelevant backgrounds. The resulting improved images are then used as the input to the YOLOv5 detection model that allows the network to be biologically oriented when extracting features and predicting the location of the object.

### Step 3: YOLO sperm cells detection

Sperm cell detection is the last point in the recommended processing image pipeline. The segmented images are loaded into a detection system known as YOLOv5^38^. Single-stage object detection that is designed to operate in a runtime way, YOLOv5 has the ability to identify objects of interest of single forward-pass mode after training with spatial characteristics. YOLOv5s was chosen in the context of the study as it has a lightweight architecture, the stable training behavior, and the ability to perform efficient detection, which makes it appropriate in the context of the microscopy-based image analysis with rather limited datasets. Despite the existence of more recent models such as YOLOv8 and YOLOv10, which do have certain improvements in architecture, YOLOv5 is a stable and affordable detector and is usually employed in biomedical image processing systems.

#### Preparing the dataset

Sperm images annotated to classify them as normal, abnormal and non-sperm objects are selected. Such tools as flipping, rotation and contrast manipulations are employed to improve the training dataset to improve generalization of model. When training, the sperm cells with normal morphology are only bound.

#### Training using YOLOv5

The YOLOv5s model structure is selected due to its high accuracy in recognising normal sperm cells since it is an economical processing structure by balancing precision and economy. The YOLOv5s (small) model version has been chosen as it is an intermediate in terms of accuracy and speed. To use transfer learning, weights of a COCO-pretrained model are also initialized. The training will be performed in batch cycle 16, learning rate of 0.001, and the YOLOv5 repository based on the PyTorch repository is optimized with Adam optimizer. The loss function is made up of objectness loss, categorization loss and localization loss (CIoU). Representing the normal sperms cells is done with only one class and abnormal or background objects are not classified in this binary representation. The Table 3 contains the training parameters.

**Table 3:** Parameters for training.

Parameter	Value
Model	YOLOv5s
Input Size	640 × 640
Epochs	150
Batch Size	16 or 32 (based on GPU)
Optimizer	Adam
Learning Rate	0.001 (with cosine scheduler)
Loss Function	Composite loss (CIoU + BCE)
Pretrained Weights	yolov5s.pt (COCO-trained)

Training is performed using the official YOLOv5 PyTorch implementation.

**Fig. 5:**
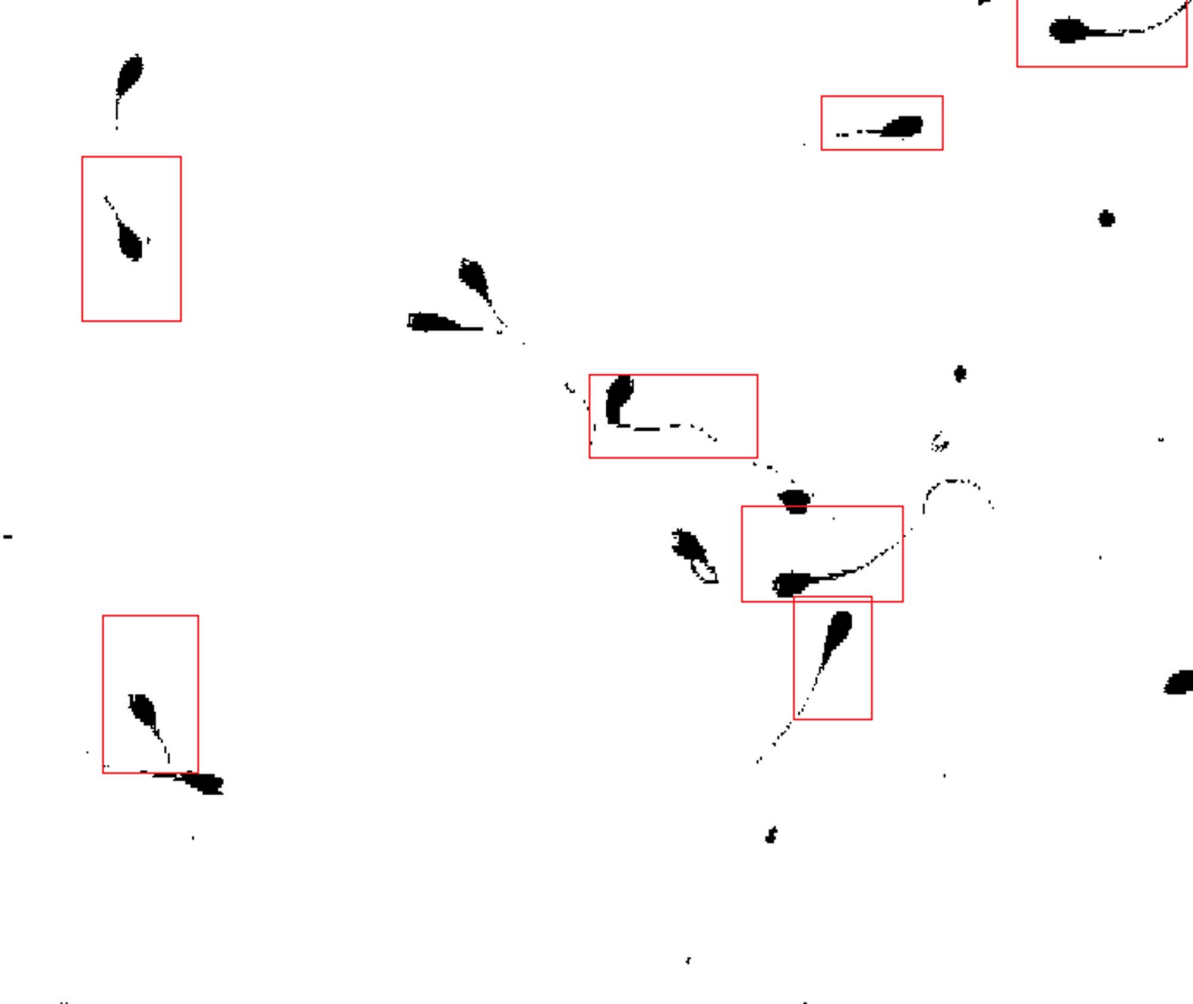
Sperm cell detected using YOLOv5.

#### Inference and post processing

YOLOv5 generates bounding boxes containing confidence scores on inference. Predictions that are considered to be normal sperm cells and exceed a user-defined confidence level (c.g., 0.5) are retained only. The use of Non-maximum Suppression (NMS) eradicates overlapping detections. This technique provides a means to distinguish morphologically normal sperm cells in complicated microscopic backgrounds to aid in additional clinical evaluation of the strengths of sperm.

#### Generation of bounding boxes of detected sperm cells

Once the sperm cells had been spotted with the YOLO object-identification framework, a bounding box was created around the identified sperm cell to identify and visualize the detection output more precisely. The bounding box is a tag as a rectangle containing all the cell sperm detected depending on the coordinates as predicted by the YOLO model.

Figure 5 illustrates the presence of sperm cells that have a bounding box. The parameters of the definition of each bounding box are four, i.e. the top-left corner coordinates (x, y)(x, y) and the dimensions (w, h) (w, h) width, height of box, respectively. These values are derived directly out of the output layer of the model which will predict the position of objects compared to the input image grid. The bounding-box is useful in defining the spatial limits of an individual sperm cell and, therefore, supports the next group of analysis like morphological measurement, tracking, or estimation of motility.

OpenCV was used with the version cv2.rectangle () to overlay the bounding boxes to the original image in order to increase its visual clarity. There was a different color code and thickness of the line that was picked so that all the boxes can be seen well without obstructing the cellular information. Also, there was optional display of the confidence scores based on the YOLO predictions along each bounding box which provided a hint of the confidence of the model in every detection.

This bounding box representation provides the intuitive display of the precision of detection and the localization and distribution of sperm cells in the sample frame. In addition, it is helpful in quantitative analysis as it allows directly calculating quantitative values of positional, morphological, and behavioral parameters of the localized sperm regions.

## Results and discussion

### Evaluation protocol

The detection of the proposed YOLO-based sperm cell detection framework was tested in a quantitative way through the comparison of automatically detectable morphologically normal sperm cells and a manual annotation ground truth dataset. The domain experts were used to produce the ground truth annotations using the standard criteria when doing the sperm morphology assessment.

Detection metrics which are universal in object detection methods such as precision, recall and F1-score were used to determine detection accuracy. Any predicted bounding box that overlapped with an annotation in groundtruth count as a true positive (TP) provided a predicted bounding box with overlap by ground truth with an Intersection over Union (IoU) threshold (greater than or equal to 0.5). FalsePositives (FP) and FalseNegatives (FN) were classified as false detections and missed detections respectively.

### Detection performance on a macro level

According to the test dataset analysis, one found the following values of the confusion matrix and given in Table 4.

**Table 4:** Confusion matrix values.

Metric	Count
TruePositives (TP)	920
FalsePositives (FP)	80
FalseNegatives (FN)	110

With the help of these values, performance measures were calculated and provided in Table 5.

**Table 5:** Overall detection performance of the proposed framework.

Metric	Value
Precision-value	0.92
Recall-value	0.89
F1-score-value	0.90

The close accuracy means that the framework suggested is effective in reducing the false classification of abnormal structures or background artifacts as healthy sperms. Simultaneously, the high recall value implicates the positive success of this model to identify most of the morphologically normal sperm cells in the microscopic images.

### Effect of improved image and segmentation

The performance improvements were greatly due to the integration of improving image and segmentation before the detection of the objects with the help of YOLO. Gaussian and Wiener filtering minimized noise and yet maintained fine morphological information that is needed to represent sperm heads and tails. The objective fidelity measures (Peak Signal to Noise Ratio, MSE and entropy values) proved the impartiality of the Wiener filtering to images with additive noise.

In addition, adaptive Havrda-Charvat entropy thresholding segmentation showed better foreground-background classification than classical Otsu thresholding especially in the case of uneven illumination and low contrast in an image. As observed in Table 2, the adaptive entropic approach provided better entropy and less RAE error hence resulted in more distinct delineation of sperm structures to feed into the YOLO detector.

This background clutter strategy minimized the background clutters and strengthened the discriminative features learnt by the YOLOv5s model hence improving the detection strength.

### Strength in the different illumination conditions

In order to measure robustness, the test pictures were grouped in terms of illumination conditions, which comprise of normal lighting, low illumination and high illumination conditions. In Table 6, the outcomes of the detection in such conditions are discussed.

**Table 6:** Performance under different illumination conditions.

Illumination condition	Precision	Recall	F1-score
Normal lighting	0.93	0.91	0.92
Low illumination	0.88	0.85	0.86
High illumination	0.90	0.87	0.88

Though a moderate decrease in performance was experienced when operating under low-light conditions, the proposed framework remained highly resistant to illumination variability since it yielded an F1-score of over 0.85. This strength can be explained by the joint application of adaptive thresholding and deep learning on features with the help of YOLOv5.

### Performance in morphological variations

The morphological variation in sperm cells is high, and this is a major problem with automated detection systems. The suggested framework was tested on the various morphology types; normal morphology, minor variations, and major shape variations are given in Table 7.

**Table 7:** Detection performance under the fit of the morphological variations.

Morphological category	F1-score
Normal morphology	0.93
Mild variations	0.89
Significant variations	0.84

In the findings, it is seen that the model is best utilized with the sperm cells that are found to have normal morphology. The relative loss of performance of significantly deformed sperm cell has been reported in relation to both clinical observations and other experiments previously reported performance studies, where extreme deviations in shape usually denote either deviant or indistinct visual features. Still, the level of F1-score 0.84 shows that the model is able to generalize on challenging biological variations.

### Discussion and comparative insights

The experimental findings suggest that proposed YOLOv5-based detection frameworks show high accuracy, robustness, and consistency and are better compared to the traditional image processing-based detection of sperm schemes described in the previous literature. The deep learning architecture in contrast to traditional thresholding, or edge-based models, is able to learn discriminative spatial information, thus making it dependable in detecting objects even in isolation with cluttered and noisy microscopic scenarios.

The proposed solution compared to the conventional CASA systems will be less reliant on hand-crafted functions and human intervention and will be scaled and able to detect events in real-time. Combined with high-quality preprocessing, adaptive segmentation, and detection based on the YOLO, this may generate a single system that can be actively used in clinical and research settings.

The proposed system in general offers a computationally efficient and clinically useful automated sperm cell detection system that has a high potential of getting integrated with computer-assisted semen analysis systems.

#### Effect of preprocessing on feature learning and detection

Before detection using YOLOv5, the combination of preprocessing and segmentation processes is significant to enhance the strength of the developed detection pipeline. In contrast to natural images, microscopic sperm images have a great amount of noise and background artifacts that can disrupt the ability of deep neural networks to learn. In the case of the existence of such artifacts, the model can be trained on irrelevant characteristics that lower the detection accuracy.

The suggested structure contributes to the visual separation of sperm cells to the background structures through noise reduction and adaptive segmentation before the detection phase. This preprocessing allows reducing the input data and allows the YOLOv5 model to concentrate on the morphological features of sperm cells during the training and inference of the model.

Besides, candidate region highlighting using segmentation is especially useful to identify small biological objects, when the background clutter would otherwise dominate the feature map produced by the convolutional layers. Consequently, the YOLOv5 detector is able to acquire more discriminative presentation of sperm morphologies, resulting in a higher localization and classification performance.

The experimental findings prove that the suggested preprocessing-based detection pipeline offers consistent and robust detection accuracy across different imaging parameters, meaning that the preprocessing part optimally complements the deep-learning-based detection sub-block.

## Data Availability

The dataset used in this study is publicly available at: http:/morfologia.cedai.cl/public.
